# Burden of Infectious Diseases in Mobile Migrants in Gold Mining Areas in Suriname’s Interior

**DOI:** 10.7759/cureus.75391

**Published:** 2024-12-09

**Authors:** Jeetendra K Jitan, Lotte Keikes, Euridice Irving, Stephen G Vreden

**Affiliations:** 1 Epidemiology and Public Health, Ministry of Health, Suriname, Paramaribo, SUR; 2 Department of Internal Medicine, Division of Infectious Diseases, Amsterdam University Medical Center, Amsterdam, NLD; 3 Department of Skills Education, Anton de Kom University of Suriname, Paramaribo, SUR; 4 Internal Medicine, Foundation for the Advancement of Scientific Research in Suriname, Paramaribo, Suriname

**Keywords:** gold miners, infectious and tropical diseases, mobile populations, neglected diseases, suriname

## Abstract

Introduction: Mobile migrants are subject to restricted healthcare access, which may result in the spread of certain infectious diseases. The aim of this study is to evaluate the burden of a subset of priority infectious diseases in mobile migrants in remote gold mining areas in the forested interior of Suriname.

Methods: This cross-sectional study enrolled mobile migrants in 13 study sites between January and June 2022. Participants underwent a structured interview, physical examination, and additional diagnostic procedures if applicable. Frequency tables were used to calculate the burden of disease.

Results: Among the 370 participants, weight loss (21%), coughing (21%), and fever (16%) were the most common symptoms. Six HIV infections, two cases of leprosy, and 15 cases of cutaneous leishmaniasis (CL) were detected. The prevalence of CL was 4.1%, of HIV (in age group 15 - 49 yrs) was 1.5%, and of leprosy was 0.5%. No cases of active pulmonary tuberculosis (pTB) were identified.

Conclusion: Our study revealed significant numbers of the infectious diseases examined, except for pTB. This emphasizes the urge to improve healthcare access in remote and underserved populations to prevent poor outcomes if individuals remain undiagnosed and untreated and to prevent the further spread of contagious infectious diseases among the general population.

## Introduction

Infectious diseases continue to threaten human health and often disproportionally affect underprivileged people. Undiagnosed and untreated contagious infectious diseases inevitably lead to increased spread and burden of disease. The World Health Organization (WHO) has defined several milestones with deadlines in the near future [1]. Countries have established programs to reach these milestones, but the distribution of specific diseases is not evenly spread among a country’s population, and targeted interventions remain pivotal in achieving these goals. Mobile and migrating populations present a typical example of people who tend to be underserved due to challenges in healthcare access [2]. These populations are identified by the WHO and the International Organization of Migrants (IOM) as a group that requires special attention [3].

Suriname is a multi-ethnic low middle-income country located in the Guiana shield area of South America, with an estimated population in 2022 of 618,040 people divided between urban (409,804) and rural (208,236) areas, including the forested interior (82,700) [4]. Primary healthcare for the urban and coastal rural population is provided by private and governmental clinics. The coastal region has seven general hospitals and some specialized medical centers and clinics.

There are numerous villages of indigenous and tribal populations in the interior of the country that are quite remote from the health facilities in the capital. Medical Mission Primary Health Care, a non-governmental organization with 58 clinics in or near the villages of indigenous and tribal populations in the country’s interior, provides healthcare for these populations. These clinics are managed by health assistants (GZAs), who received formal, Government-approved training of four years prior to deployment. The GZAs are supervised remotely by medical doctors who also regularly make supervisory visits. The services provided in these clinics include health promotion, primary health care, antenatal care, vaccination, and dental care. The permanent residence and availability of GZAs, a well-organized logistic system (transportation by car, boat, and airplane) for emergencies and cases that need secondary care, ensure basic health care, albeit less than in urban settings, for the indigenous and tribal populations in Suriname [5,6].

The population in the forested interior of Suriname working in gold mining and logging consists of approximately 20,000 people. The majority of this population is foreign and of Brazilian origin and does not reside in the villages, but in dwellings that arise and disappear, depending on the activities, mainly artisanal small-scale gold mining [7]. The mobility of these populations is not limited to one country, since many are crossing borders of neighboring countries, mainly French Guiana, where they illegally work in artisanal small-scale gold mining. As part of Suriname’s malaria elimination efforts, a system of malaria service deliverers has been set up in the mining areas, existing of people recruited from these communities, after training, provide malaria diagnosis and treatment free of charge [8].

The implementation of the Malaria Service Deliverers (MSDs) network, has significantly contributed to the elimination of locally transmitted malaria in the artisanal and small-scale gold mining (ASGM) community, enabling the Ministry of Health to request for WHO’s certification of malaria elimination by the year 2025. MSDs in the area bordering French Guiana are also involved in the MALAKIT project, which distributes self-diagnosis and treatment kits to transborder gold-mining migrants. This project reduced the number of imported malaria cases from French Guiana with 76.3% in 2020 [9].

Migrating populations have only access to healthcare if they move, at their own expense, to a rural or urban health facility. Mobile populations tend to postpone medical care and follow-up consultations, even when seriously ill, because of high traveling costs and expected loss of income [7].

We hypothesize that mobile underserved populations are at more than average risk of harboring undiagnosed infectious diseases other than malaria. The present study aims to assess the burden of a prespecified subset of infectious diseases (HIV, pulmonary tuberculosis, cutaneous leishmaniasis, and leprosy) in mobile migrants in the remote gold mining areas in Suriname.

## Materials and methods

Study design and population

This study has a cross-sectional design consisting of a structured interview, and physical examination followed by diagnostic procedures, all focusing on four infectious diseases: HIV, pTB, CL, and leprosy. The study population included people residing in remote ASGM areas in the forested interior of Suriname.

**Figure 1:**
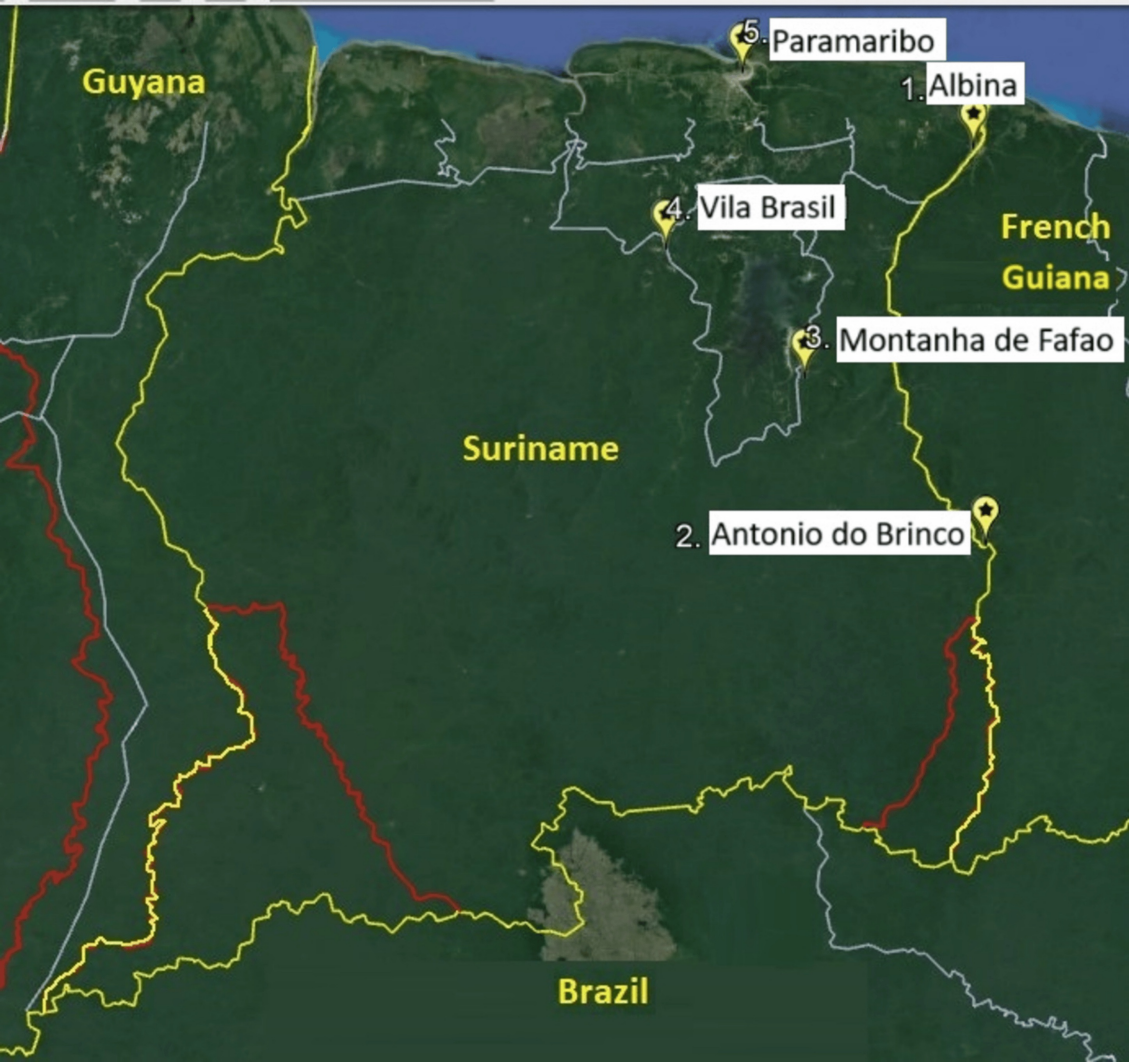
Geographic locations of the survey regions

The study was performed in four gold mining regions in the interior of Suriname, where a weak infrastructure and long distances to healthcare services are unfavorable factors for healthcare access (Figure 1). A total of 13 study sites were included in these regions, in the east of the country along the Maroni River in the settlements Antonio do Brinco/Ronaldo and Albina and in the middle of the country the settlements of Alimoni (Lake area) and Villa Brazil (Upper Saramacca River). In parallel to what has been proven useful in the Malakit study, an additional research site was organized at a hotel in the capital of Suriname, Paramaribo, which collaborates with the Trop Clinic. This last selected site is often visited by mobile migrants, so those who had missed the initial survey at the site in the interior where they normally stay were thus offered another opportunity to participate [9]. Random selection of participants in the field was practically not attainable, and therefore all people were invited to participate. Inclusion criteria were that participants needed to be residing in a mining area and prepared to sign the consent form. People who just happened to be around during the survey, but resided outside of the mining area, were excluded from participation. We purposely did not limit our study to adults, since children may also be affected by the diseases that were studied.

Data collection and outcome measurements

Before the actual visit to the sites, the local leadership was informed and requested to collaborate by providing a venue for the survey and informing the people about the planned visit of the study team. Upon arrival of the study team, the offered facility was prepared for the survey and posters were distributed in the community.

After obtaining informed consent (in the case of children, from a parent or guardian), a structured interview focusing on symptoms and knowledge of the four infectious diseases studied was conducted followed by counseling by a well-trained fieldworker, who was proficient in the participant’s language. Thereafter, participants were subjected to limited physical examination and additional diagnostic procedures.

After the structured interview, the entire body was inspected for signs of cutaneous leishmaniasis and leprosy. If cutaneous leishmaniasis was suspected, photographs and biopsies of lesions were taken and sent to the government dermatological service in Paramaribo for further investigation. For leprosy, the body was inspected for pale (hypopigmented) or reddish spots and subsequently tested for loss of sensation and evaluated for thickened or enlarged peripheral nerves with loss of sensation and/or weakness of the corresponding muscle(s). In the case of suspected lesions, the participant was referred to the above-mentioned dermatological services.

Participants were explicitly informed that they could opt out of HIV testing. If they agreed, the Determine^TM^ rapid diagnostic test (RDT) was used for initial HIV screening, and if a positive result was obtained, a second RDT (UniGold^TM^) was performed to confirm the result. If this second test also turned out positive, participants were counseled and referred to a hospital in Paramaribo for further management. Sputum was collected from participants who reported coughing for at least one week. Since on-site investigation of collected material was not feasible, the sputum was preserved in 70% ethanol and transported to a lab in Paramaribo, where a PCR GeneXpert analysis for TB was carried out [10].

**Figure 2:**
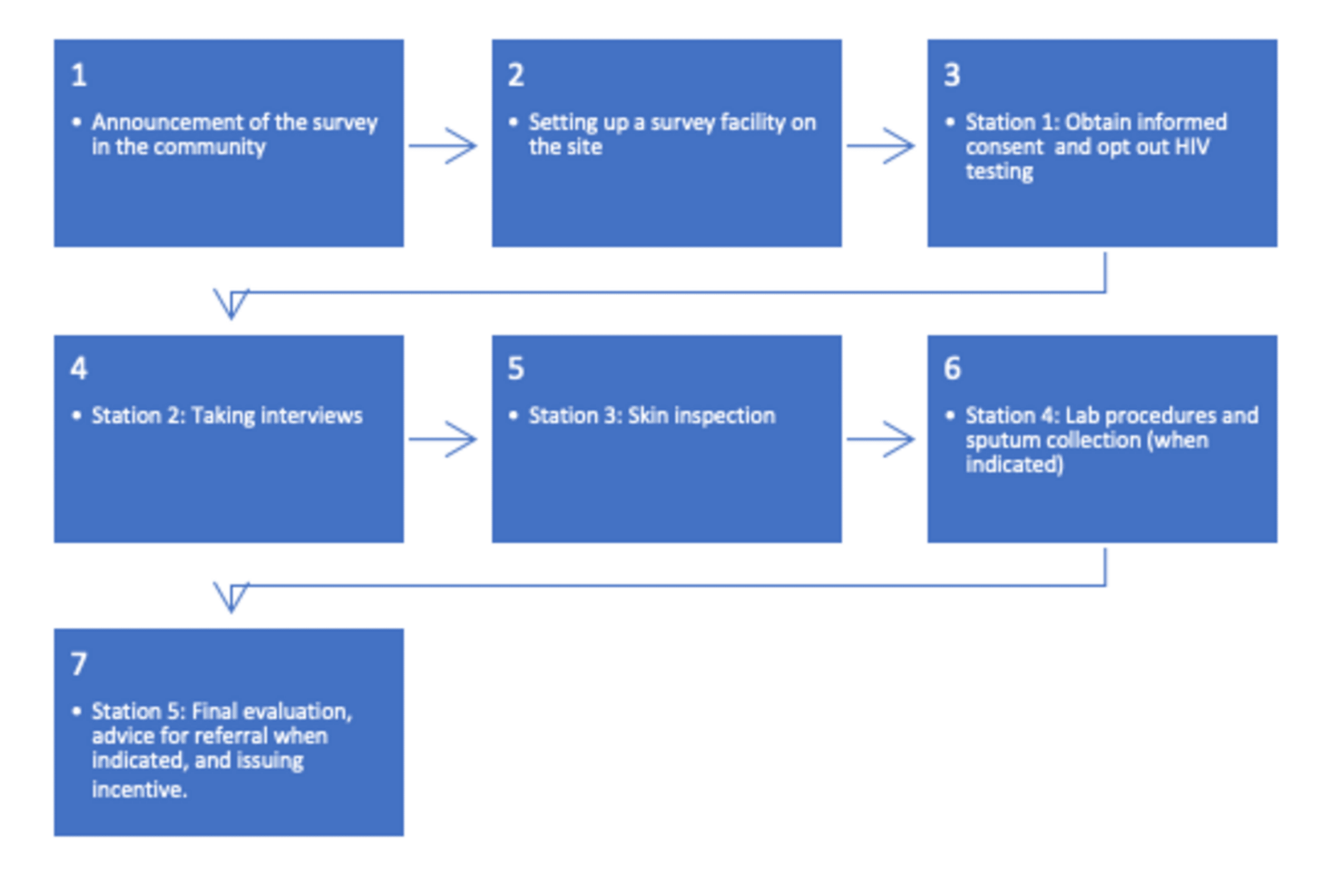
Flow chart of the study procedure Numbered stations indicate the order of procedures carried out by different fieldworkers.

After completing all on-site study procedures, participants received an impregnated bed net, telephone credit, an impregnated bed net, and condoms as an incentive. The study procedure is summarized in Figure 2. 

Statistical analyses

Study data were collected, managed, and verified on site using REDCap electronic data capture tools, hosted at the Anton de Kom University Paramaribo. REDCap (Research Electronic Data Capture) is a secure, web-based software platform designed to support data capture for research studies [11]. The results were analyzed using the IBM SPSS Statistics for Windows, Version 28.0 (released 2021, IBM Corp., Armonk, NY). Descriptive statistics of the population were calculated with frequencies and percentages. The prevalence with corresponding 95% confidence intervals (95% CI) was calculated.

Ethical approval

Each participant was asked to sign an informed consent form after providing information about the objectives of the study, the structured interview, the physical examination, and the use of biological samples for further investigation. For children under 18 years of age, consent was requested from parents or legal guardians.

The study was approved by the ethical committee (Commissie voor Mensgebonden Wetenschappelijk Onderzoek/National Ethics Committee) of the Ministry of Health in Suriname (document number: AG.4468/21).

## Results

Study population

A total of 370 participants were included in the study carried out from January until June 2022. The mean age was 43 years for men (standard deviation (SD): 13 years) and 41 years for women (SD: 11 years). The male-to-female ratio was 2:1 and the vast majority of the population had a Brazilian origin (81%). An overview of the baseline characteristics is presented in Table 1. Most participants originated from the Upper Lawa (32%), Upper Lake (27%), and Upper Saramacca (32%) areas (Table 2). Of the 81% (n = 299) Brazilian participants, the majority originated from Maranhão (n = 166; 56%) and Pará (n = 69; 23%).

**Table 1:** Baseline characteristics of the study population

		Total population
		n = 370 (100%)
Subjects	Results	n (%)
Gender	Female	125 (34)
	Male	245 (66)
Age categories (years)	≤20	20 (5)
	21-30	55 (15)
	31-40	90 (24)
	41-50	98 (27)
	51-60	80 (22)
	>60	27 (7)
Country of origin	Suriname	44 (12)
	Brazil	299 (81)
	Dominican Republic	11 (3)
	Cuba	5 (1)
	China	4 (1)
	Other	10 (3)
Length of stay in Suriname (years)	<1	53 (14)
	1-5	74 (20)
	6-10	66 (18)
	11-15	51 (14)
	16-20	44 (12)
	21-25	23 (6)
	26-30	8 (2)
	>30	7 (2)

**Table 2:** Distribution of participants according to study areas and sites

Study area	Study sites (number of sites)	Gender		Total
		Women	Man	
		n (%)	n (%)	n (%)
Marowijne	Albina (1)	8 (2)	5 (1)	13 (4)
Upper Lawa	Antonio do Brinco (2)	45 (12)	72 (19)	117 (32)
Upper Lake	Alimoni (4)	25 (7)	76 (21)	101 (27)
Upper Saramacca	Villa Brazil (5)	39 (11)	81 (22)	120 (32)
Paramaribo	Parama (1)	8 (2)	11 (3)	19 (5)
Total		125 (34)	245 (66)	370 (100)

Burden of symptoms and prevalence of infectious diseases

The participants were asked about symptoms and complaints in relation to the infectious diseases studied. Weight loss (21%), coughing (21%), and fever (16%) were most commonly reported. Diagnostics were routinely performed for HIV, cutaneous leishmaniasis and leprosy, and pulmonary TB in case of coughing for more than one week (Table 3).

**Table 3:** Overview of symptoms reported by the participants

		Total population
		n = 370 (100%)
	Answers	n (%)
Fever	No	310 (84)
	Yes	60 (16)
Night sweats	No	339 (92)
	Yes	31 (8)
Appetite	Good	339 (92)
	Bad	31 (8)
Weight loss	No	292 (79)
	Yes	78 (21)
Coughing	No	293 (79.2)
	<1 week	74 (20)
	>1 week	3 (0.8)
Skin abnormalities	No	322 (87)
	Yes	48 (13)

While most participants agreed to undergo HIV testing (n=363; 98%), a few (n=7; 2%) opted out. Six participants, all of Brazilian origin, tested positive (1.7%; 95% CI: 0.8-3.6%), of which one had been previously diagnosed (six years prior) and was already on treatment. Three individuals who tested positive were in the age group 15-49 years, and therefore, the prevalence among Brazilian participants in this age group was 1.5% (95% CI: 0.5%-4.3%). In the age group > 50 years, the HIV prevalence among Brazilian participants was 3.1% (95%CI: 1.1%-8.7%). Three out of 77 patients reported coughing for more than one week. In these patients, sputum samples were analyzed for pulmonary TB tested negative. A total of 15 cases of CL (4.1%; 95% CI: 2.5-6.6%) were identified. Five active cases (1.4%) of CL and another 10 cases (2.7%) with a recent infection were detected in the Upper Lawa region, in the Antonio do Brinco/Ronaldo site. All of these were already on treatment, either in a clinic in French Guiana or by self-injection of illegally purchased pentamidine. Leprosy was detected in two (0.5%; 95% CI: 0.1-0.9%) unrelated cases. One had not been previously diagnosed and the second one was a defaulter.

Knowledge population regarding infectious diseases

Most participants knew what HIV was (95%) and the majority (66% (n = 244)) had been tested for HIV in the past. One participant tested positive for HIV in the past, and six participants had an HIV-positive partner in the past. Most participants were aware of the availability of HIV treatment (92%). For leprosy, 79% (n = 293) of the participants knew what leprosy was and 20% (n = 74) knew someone affected with leprosy, and of this group, 21 participants (28%) had been in close contact with someone with leprosy.

## Discussion

In our study, we identified a significant prevalence of HIV, cutaneous leishmaniasis, and leprosy in a sample of mobile migrants in gold mining areas in the forested interior of Suriname.

The HIV prevalence among the Brazilian participants in the age group 15 - 49 years was 1.5% (n=6). This is higher than the estimate for the Caribbean (1.2%) and for Brazil (0.6%) [12]. This figure is most likely not applicable to all states in Brazil; a report from 2023 shows that both Maranhão and Pará, where the majority of participants originate from, had HIV detection rates in 2022 that were more than twice the national average [13]. We found a 3% prevalence of HIV infections in people older than fifty years. This corroborates with findings that the majority of HIV-infected persons are now over fifty years old and that this age group significantly contributes (16.3%) to the number of new infections [14].

No cases of active pulmonary TB were identified, which is in line with our expectations, given the limited sample size of our study, even with the relatively high incidence rates and upward trend of pulmonary TB in Brazil (29.7 and 45 per 100.000 people in Maranhão and Pará respectively, between 2011 and 2019) [15,16]. 

Cutaneous leishmaniasis is highly prevalent in the Amazonian Forest [17,18]. Our study identified a prevalence of 4.1%, which is higher than the reported 2.4% in a previous study conducted in French Guiana in 2019 among 380 Brazilian gold miners, but lower than the 8.3% that was reported among the same population in 2015 in 421 gold miners (93.8% were Brazilian) [3,19]. The incidence rate of CL infections seems to fluctuate depending on the dry or rainy season, which may explain the differences in prevalence and reported seasonal occurrence [20].

Our study identified two cases of leprosy (0.5%), one case originated from Suriname and the other from Brazil. Studies performed in 2019 in gold mining areas in French Guiana (n = 380) reported a prevalence of 0.8% [21,22]. The highest rates in Brazil are found in the states of Amazonas, Maranhão, and Pará [23]. A study conducted between 2010 and 2015 in Brazil showed a prevalence of 1.42/10,000 with a general detection coefficient of 15.44/100,000 inhabitants [24]. Although the prevalence in our study is lower than the previous study in French Guiana, we conclude - since the majority of this population is Brazilian - that our prevalence is much higher than the estimated prevalence of 0.011% in Brazil. Brazil is among the countries with the highest prevalence of leprosy, and therefore, finding one case in this relatively small sample is not surprising [25].

Our study has some limitations. The data presented might not fully represent the study population since the setting did not allow for a random selection of participants. We invited all people present in the included areas and enrolled all available and eligible participants. This may have caused selection bias in different ways. First, some people who were willing to participate did not do so, since they could not afford to suspend their work for financial reasons (no work and no pay) in the limited period the research team was present. Furthermore, we experienced that the participants had preferential attention for screening and management of non-communicable diseases (e.g., heart disease, diabetes, and cancer) instead of infectious diseases, which could have been of influence on participation preparedness. Lastly, a selection of the numerous gold mining regions in the country was included in our study, which may limit the generalizability.

To the best of our knowledge, this is the first study that evaluated the prevalence of this selection of infectious diseases in Suriname’s mobile and migrant population. The relevance of the study is emphasized by the ongoing gold rush in the country and beyond, leading to an influx of mainly Brazilian gold miners that has been going on for approximately 25 years [3,24] and is still rising given the significant number of people who arrived in less than one year (n = 53; 14%). The mobility of this population in Suriname’s interior is not only characterized by their migration to the country, but also by a high rate of in-country mobility. A study by Heemskerk et al in the years 2019-2020, reported that 44% had at least once changed their working location in the previous 2 years, and 20.5% even did so for three to ten times. The motivation for moving is often the expectation of better profits [7]. The gold miners are a vulnerable population, because of the remoteness of their work resulting in limited or no health care access, poor sanitary circumstances, and their close contact with each other and potential vectors of diseases (e.g., mosquitoes and sandflies) in the rainforest. The migrating aspect of their work facilitates the further spread (contagious) infectious diseases to the general population.

For malaria, the Surinamese Ministry of Health has developed a system responsible for the information, diagnosis, and treatment of malaria since 2006. This Malaria Program is carried out by people recruited from the mining communities, called Malaria Service Deliverers, who undergo special training and are provided with educational materials, malaria RDTs, and malaria medication. They also prepare blood smears, which are stained and evaluated at the lab in the capital Paramaribo for verification of the results obtained by the rapid test.

This innovative approach to malaria control in remote populations was implemented bn the Malaria Program of Suriname, and was awarded the Malaria Champions of the Americas Award in 2010, resulting in the successful elimination of malaria in Suriname [26]. For other (infectious) diseases, there are currently no services available in the mining fields. Our suggestion would be to expand the tasks of MSDs after additional training. Building on the successful implementation of MSDs in reducing malaria could be a valuable intervention to alleviate the burden of other infectious diseases but also non-infectious diseases (such as hypertension and diabetes) of vulnerable populations in remote areas.

## Conclusions

Based on the findings of our study, we conclude that mobile populations, such as migrants in the remote gold mining areas in the interior of Suriname, bear a significant burden of infectious diseases studied, except for pulmonary TB. The observation that about one in seven participants in this survey arrived in the mining fields less than one year before indicates that the influx of new miners is ongoing, most likely fueled by the soaring gold price. This finding further emphasizes the necessity to address the problem of poor accessibility to healthcare in these populations. It is worrying for individual health outcomes if chronic infectious diseases remain undiagnosed and untreated, but another concern is the risk of further spread, warranting interventions to improve access to health care in remote areas in Suriname and beyond. Improvement of health access should not only be focused on infectious diseases, since, worldwide, there is also an epidemic of noncommunicable diseases. The feasibility of merging the very limited healthcare provision of the migrant populations by malaria service deliverers with the services provided by medical missions to the stable communities in the interior should therefore be assessed.
